# Psychiatric consequences in hospitalized patients affected by COVID-19 (RECOVER-PSY)

**DOI:** 10.1192/j.eurpsy.2023.505

**Published:** 2023-07-19

**Authors:** F. Wiedenmann, G. Fior, E. Bergamelli, R. Del Giudice, O. Gambini, A. D’Agostino

**Affiliations:** ^1^ Università degli Studi di Milano; ^2^Mental Healt Department, ASST Santi Paolo e Carlo, Milano; ^3^Mental Healt Department, ASST Ospedale Papa Giovanni XXIII, Bergamo; ^4^Mental Healt Department, Università degli Studi di Milano, Milano, Italy

## Abstract

**Introduction:**

COVID-19 had a significant impact on the mental health of the affected population. Such multifactorial risk for a deterioration of mental health suggests the need to identify groups of patients with psychiatric vulnerability and to establish strategies of intervention based on scientific evidence.

**Objectives:**

The aim of the study was to identify psychiatric outcomes one year after recovery and possible associations between these and the clinical, anamnestic, and sociodemographic variables.

**Methods:**

The Mini International Neuropsychiatric Interview was employed to assess current and lifetime mental illness in a cohort of 100 patients discharged between March and April 2020 from COVID-19 wards of the San Paolo Hospital in Milan, Italy. The Kendall rank correlation coefficient was administered to measure the ordinal association between clinical-demographic variables and the psychiatric diagnoses of patients. Bivariate correlation was used to explore the association between psychiatric outcomes and the sample characteristics.

**Results:**

Almost one third of subjects screened positive for a diagnosis of a new psychiatric disorder, and a novel onset of psychiatric morbidity did not differ significantly in patients with and without a positive history of mental illness (42 and 58%). New psychiatric disorders were grouped into stress reactions, anxiety-group disorders and mood disorders. Concerning demographic characteristics, advanced age represented a protective factor against the onset of new psychiatric disorders (rτ = -0,203, p =0,008). Despite a lower risk of contracting the infection, women in our cohort were more vulnerable to psychiatric post-Covid symptoms (rτ =0,190, p =0,029). The correlation between the onset of new psychiatric disorders and some pre-admission vulnerability factors, such as an overweight condition (rτ =0,185, p =0,026) and a positive medical history for cigarette smoking (rτ =0,203, p =0,026), were statistically significant. Moreover, subjects who reported taking a therapy to control the infection prior to hospitalization were more likely to receive a new psychiatric diagnose (rτ =0,269, p =0,005). Of note, variables related to the severity of hospitalization such as oxygenation intensity, days of hospitalization, or requirement of intensive car were not associated with new psychiatric diagnoses.

**Conclusions:**

The onset of psychiatric disorders shows a relevant frequency in patients hospitalized for COVID-19, suggesting that mental health services should structure adequate screening and diagnosis methods. Three levels of intervention can also be expected to reduce the overall risk and burden of psychiatric morbidity: increasing awareness regarding modifiable risk factors; guaranteeing a minimal level of mental health support to patients hospitalized for COVID-19; providing personalized interventions with respect to gender and age groups.

**Disclosure of Interest:**

None Declared

